# Changes in Health State Utility Values in Japanese Patients with End-Stage Breast Cancer

**DOI:** 10.3390/curroncol28050356

**Published:** 2021-10-18

**Authors:** Tsuguo Iwatani, Shinichi Noto, Koichiro Tsugawa

**Affiliations:** 1 Department of Breast Surgery, National Cancer Centre Hospital East, 6-5-1 Kashiwanoha, Chiba 277-8577, Japan; 2 Department of Breast and Endocrine Surgery, St. Marianna University School of Medicine, 2-16-1 Sugao Miyamae, Kawasaki 216-8511, Japan; koitsuga@marianna-u.ac.jp; 3 Department of Health Sciences, Niigata University of Health and Welfare, 1398 Shimami-cho, Kita-ku, Niigata 950-3198, Japan; noto@nuhw.ac.jp

**Keywords:** health state utility values, breast cancer, Japanese patients, clinical oncology, HSUV

## Abstract

We aimed to determine the dynamic trends in health state utility values (HSUVs) in patients with end-stage breast cancer. We selected 181 patients comprising 137 with primary breast cancer (PBC) and 44 with metastatic breast cancer (MBC) (28 survivors and 16 patients with MBC death). HSUVs were 0.90 and 0.89 in patients with PBC and 0.83 and 0.80 in those with MBC (survivors) at 6 and 3 months, respectively, before the end of the observation period; these values were 0.73 and 0.66, respectively, in those with MBC (deceased) during the aforementioned period. The root-mean-squared error (RMSE) for the decrease in HSUVs over 3 months was 0.10, 0.096, and 0.175 for patients with PBC, MBC (survivors), and MBC (deceased), respectively. One-way analysis of variance for differences in absolute error among the groups was significant (*p* = 0.0102). Multiple comparisons indicated a difference of 0.068 in absolute error between patients with PBC and those with MBC (deceased) (*p* = 0.0082). Patients with end-stage breast cancer had well-controlled HSUVs 3 months before death, with a sharp decline in HSUVs in the 3 months leading up to death.

## 1. Introduction

Breast cancer is the most frequently diagnosed malignant disease. In 2020, it was the leading cause of cancer death, with approximately 2.3 million new diagnoses and 685,000 deaths worldwide [1]. In Japan, female breast cancer has the highest incidence, with 91,605 new cases reported in 2017 and 14,839 deaths reported in 2019 [2]. Breast cancer is characterised by three major stages as follows: early stage (primary breast cancer (PBC)), metastatic and recurrence stage (metastatic breast cancer (MBC)), and terminal stage. While the treatment goal of PBC is to cure the disease, that of MBC is to prolong the survival time and maintain good health-related quality of life (HRQOL). 

The advent of innovative drugs has improved survival in breast cancer; however, it reportedly increases healthcare costs [3,4,5]. Moreover, the cost of healthcare under public health insurance is increasing every year [3]. Japanese medical expenses are also increasing owing to medical innovations. Thus, the Japanese government introduced a health technology assessment (HTA) in April 2016 [6]. HTA is a systematic evaluation process of the scientific value, economic, social, and ethical issues related to medical technology, with fair and robust methods while ensuring transparency. The aim of HTA is to provide information to create efficient and safe medical policies for patients to achieve an optimal value. HTA includes both the cost-effectiveness and the relative utility of comparable treatment methods (drugs) and their social/ethical value. HTA decision making is strongly influenced by the results of medical economic evaluations, such as cost-effectiveness analyses [7]. 

The Official Guideline for the Economic Evaluation of Drugs/Medical Devices in Japan recommends the use of quality-adjusted life years (QALYs) as a basic outcome in the guidelines for the outcome measure of Japanese HTA. In the QALY method, quality adjustment is based on a set of values termed health state utility values (HSUVs), which suggests the relative desirability of the health condition. These utilities reflect the value of health state and improvement in health conditions. HSUVs are a measure of preference-based HRQOL and represent an individuals’ preference for being in a particular health state. Moreover, they are anchored on a 0 (dead) to 1 (full health) scale, with negative values representing health states worse than death. The Japanese Guideline for Preparing Cost-Effectiveness Evaluation to the Central Social Insurance Medical Council recommends that HSUVs should be reflective of the value of the general population (using a preference-based measure (PBM) or direct methods, such as the standard gamble (SG) and the time trade-off (TTO)) while calculating QALYs. Moreover, the use of PBMs with a value set developed in Japan using TTO (for example, the EuroQol 5-Dimension 5-Level (EQ-5D-5L) questionnaire [8]) is recommended as the first choice while collecting new Japanese HSUVs for cost–utility analyses [9].

There have been several reports on HSUVs in patients with breast cancer [10,11,12,13,14,15,16,17,18,19,20,21]. Researchers have reported on various aspects, including review articles [10,11,17,18], studies on health care providers [12], studies on the extent of disutility due to adverse events of anticancer drugs [13,14,19], studies focused on age [15], and studies focused on national and ethnic characteristics [16,20,21]. Differences in HSUVs according to the clinical status of breast cancer are important. Moreover, it is imperative to examine HSUVs according to cancer stage [22] and line of treatment [17]. In particular, there are few reports on HSUVs in patients with end-stage breast cancer. Earle et al. reported HSUVs ranging from 0.16 to 0.54 in such patients [10]. Paracha et al. reported HSUVs ranging from 0.514 to 0.756 in patients with end-stage cancer [17]. Thus, HSUVs of patients with end-stage breast cancer vary widely, and their wide range contributes to the inadequacy of point estimates of HSUVs alone.

Therefore, the aim of this study was to determine the dynamic trends in HSUVs in patients with end-stage breast cancer.

## 2. Materials and Methods

### 2.1. A Prospective Cohort Database of HSUVs for Japanese Breast Cancer Patients

We developed a prospective cohort database of HSUVs for Japanese patients with breast cancer, which was linked to their social background and treatment history. The study sample included patients who attended the outpatient breast clinic at the Department of Breast and Endocrine Surgery, St. Marianna University School of Medicine, Kawasaki, Japan, between May 2016 and September 2018. Patients were consecutively sampled during the principal investigator’s outpatient clinic days, and patients who gave written consent to participate in the study were enrolled. The inclusion criteria were as follows: (i) Japanese women aged >20 years, (ii) a histopathological diagnosis of breast cancer, and (iii) provision of written informed consent for study participation. The exclusion criteria were as follows: (i) undergoing active treatment for mental disorders and (ii) participation in other clinical trials. This study was registered with the University Hospital Medical Information Network (UMIN) Clinical Trials Registry, managed by the National University Hospital Council of Japan (UMIN 000022517). The study protocol was approved by the Institutional Review Board and Ethics Committee of St. Marianna University School of Medicine and was performed in accordance with the ethical standards as mentioned in the 1964 Declaration of Helsinki and its later amendments. All participants were informed of the purpose and methods of the study and provided their written informed consent to participate.

We developed the study schedule based on patients’ disease conditions and treatment. Patients’ social background (education level, marital status, residential environment, employment status, and household income), breast cancer condition, and HSUVs (measured using the EQ-5D-5L questionnaire) were examined at entry, according to the study schedule.

Patients with PBC who were being followed up were surveyed every 24 weeks (±2 weeks) using a QOL survey questionnaire. Those with PBC receiving hormone therapy were surveyed every 12 weeks (± 2 weeks) using a QOL survey questionnaire and a PRO survey of adverse events because of hormone therapy. We surveyed those with PBC receiving chemotherapy and on adjuvant molecular targeted therapy every 3 weeks (± 1 week) and 9 weeks (± 1 week), respectively, using a QOL survey questionnaire. In patients with PBC receiving radiation, a one-time QOL survey was performed during the treatment period. Patients with MBC on hormone therapy were surveyed every 9 weeks (± 3 weeks) using a QOL survey questionnaire. Patients with MBC receiving chemotherapy and on molecular targeted therapy were surveyed every 6 weeks (± 3 weeks) using a QOL survey questionnaire. In addition, we performed a one-time QOL survey during the treatment period in patients with MBC receiving radiation. The survey was conducted in the privacy of the patients. Research assistants distributed the questionnaires to the participants before the physician’s examination and collected them approximately 30 min after. After collecting the questionnaires from the patients, research assistants checked the responses and asked the patients to complete any missing items. All data were collected in a similar manner, according to the manufacturer’s protocol. There were no missing data for the repeated measures.

### 2.2. Preference-Based Measurement for Calculating HSUVs

The EQ-5D-5L is a preference-based measurement scale developed by the EuroQol groups [8]. This descriptive system comprises five dimensions as follows: mobility, self-care, usual activities, pain/discomfort, and anxiety/depression. Each dimension has the following five levels: no problems, slight problems, moderate problems, severe problems, and extreme problems. This questionnaire defines 3125 health state patterns, ranging from 11,111 (representing the best health state) to 55,555 (worst health state). These 3125 health state patterns may be converted into a country-specific single index value (HSUVs) using country-specific value sets, which have been derived from large country-specific validation studies using time trade-off/discrete choice methodology. Moreover, they anchor 1 for ‘perfect health’ and 0 for ‘dead’ [10]. In other words, HSUVs calculated using the EQ-5D-5L derived from the Japanese value set represent a value of the respondent’s health status from the general Japanese perspective.

### 2.3. Transition in HSUVs in Japanese Patients with Breast Cancer

As this study aimed to identify dynamic trends in HSUV in patients with end-stage breast cancer, we decided to compare patients according to their survival status and disease state; thus, we classified them into end-stage and non-end-stage patients.

**Figure 1:**
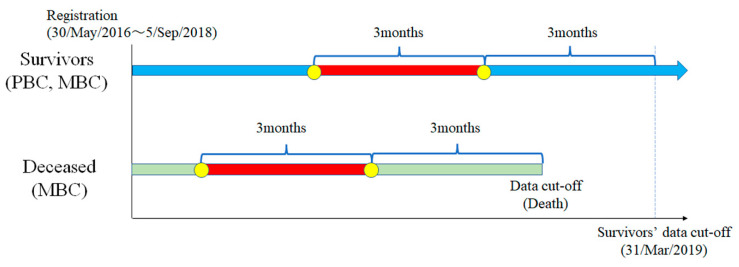
Study design: PBC, primary breast cancer; MBC, metastatic breast cancer.

We followed up with the patients enrolled in the prospective cohort database between 30 May 2016 and 5 September 2018 until 31 March 2019. For the surviving patients, we included HSUVs from two survey points at 6 and 3 months before commencing from the date of the last data collection. For deceased patients, we included HSUVs from two survey points at 6 and 3 months before the date of death (Figure 1). We classified the patients into four groups—namely, PBC survivors, PBC deceased, MBC survivors, and MBC deceased. We excluded PBC deceased from the study because they died of causes other than breast cancer. Of the three groups, PBC survivors, MBC survivors, and MBC deceased comprised the major group of patients because it included data from those who died with end-stage breast cancer. We analysed PBC and MBC survivors as the control groups.

### 2.4. Statistical Analyses

We determined the root-mean-squared error (RMSE) of the difference among the mean HSUVs of the aforementioned three groups 6 and 3 months before the last data collection date (or death). We examined the null hypothesis ‘absolute error of HSUVs is equal in the three groups’, using the one-way analysis of variance. A *p*-value < 0.05 (typically ≤0.05) was considered statistically significant. We performed multiple comparisons (Tukey’s test) to determine the group that differed from others, following the rejection of the null hypothesis. We studied the trend of HSUVs in patients with end-stage breast cancer 6 months before death, 3 months before death, and 0 at death using a parallel plot. Statistical analyses were performed using JMP statistical software version 15 (SAS Institute Inc., Cary, NC, USA).

## 3. Results

**Figure 2:**
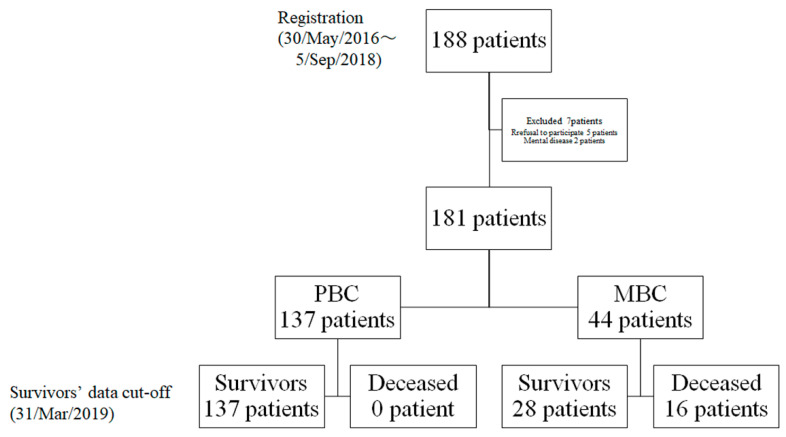
Flowchart depicting patient distribution: PBC, primary breast cancer; MBC, metastatic breast cancer.

The study included 181 patients, comprising 137 with PBC and 44 with MBC enrolled between 30 May 2016 and 5 September 2018 (Figure 2). The cut-off date was 31 March 2019. Data collection was continued in 28 patients with MBC on 31 March 2019. It was discontinued in 16 patients with MBC who died of breast cancer. Table 1 and Table 2 summarise their clinical characteristics.

**Figure 3:**
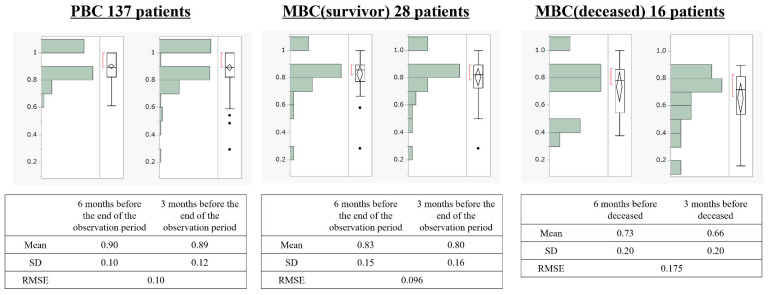
Transitions of health state utility values in the three groups: MBC, metastatic breast cancer; PBC, primary breast cancer; RMSE, root-mean-squared error; SD, standard deviation. Black dots indicate outliers.

**Figure 4:**
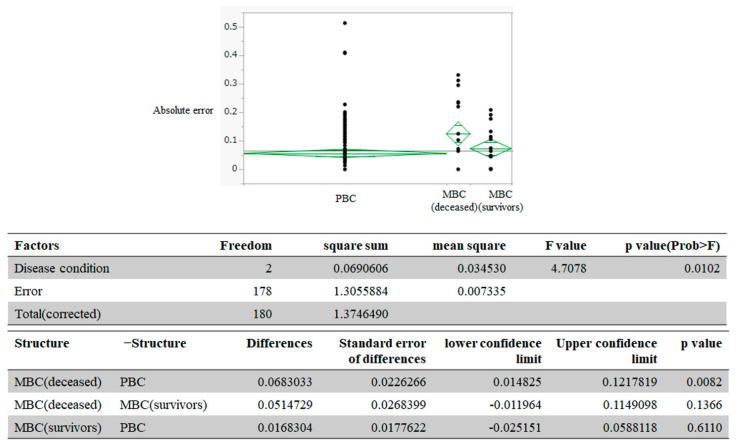
Examination of between-group differences of health state utility values: MBC, metastatic breast cancer; PBC, primary breast cancer. Black dots indicate each data of absolute error.

Figure 3 depicts the transitions of HSUVs in the groups. In patients with PBC, HSUVs were 0.90 at 6 months and 0.89 at 3 months before the end of the observation period. In MBC (survivors), HSUVs were 0.83 and 0.80 at 6 and 3 months before the end of the observation period; in MBC (deceased), these values were 0.73 and 0.66, respectively. The RMSE for the decrease in HSUVs over 3 months was 0.10 for PBC, 0.096 for MBC (survivors), and 0.175 for MBC (deceased). A one-way analysis of variance for the difference in absolute error among the groups demonstrated *p* = 0.0102 (Figure 4). We performed multiple comparisons using Tukey’s test to determine the groups that differed in absolute error. We observed a difference of 0.068 in absolute error between patients with PBC and those with MBC (deceased) (*p* = 0.0082).

**Figure 5:**
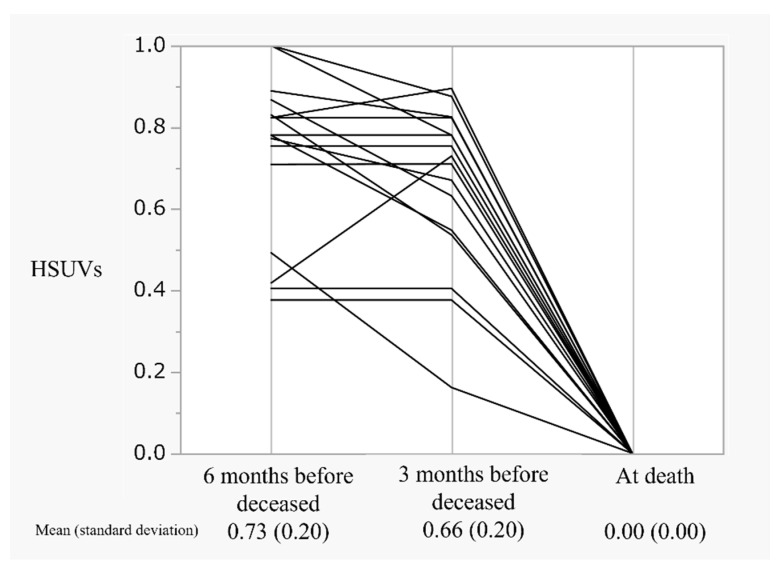
The trend of HSUVs in patients with end-stage breast cancer: HSUVs, health state utility values.

Figure 5 displays the parallel plot of HSUVs in patients with end-stage breast cancer, 6 months before death, 3 months before death, and 0 at death. HSUVs measured 6 and 3 months before death were 0.73 and 0.66, respectively, as HSUVs defined death as 0.

## 4. Discussion

QALY is a measure of effectiveness during the economic evaluation of medical interventions and is a single number that multiplies the gain in quantity (increase in the years of survival) with the improvement in quality (improvement in HRQOL). HSUVs are based on health state preferences and are expressed as a unidimensional number, with perfect health and death being 1.0 and 0, respectively. Paracha et al. reported that HSUVs in patients with MBC decrease with treatment progress [17]. In contrast, no researchers have dynamically examined the transition of HSUVs from late line treatment to death in patients with terminal stage breast cancer by following the time series. Therefore, we investigated the dynamic trends of HSUVs in patients with end-stage breast cancer.

First, we compared the evolution of HSUVs in the aforementioned three groups over 3 months. This is the first report to describe the time-dependent decline in HSUVs in patients with end-stage breast cancer, shortly before their death. Haslam et al. reported that most clinical trials in oncology assessed the QOL during treatment or intervention, as well as during the prescribed follow-up period, but rarely assessed the QOL for disease progression until the end of the patient’s life [23].

Figure 5 also depicts the predicted trend of HSUVs in patients with breast cancer at the end of life. These patients demonstrated well-controlled HSUVs of 0.66, 3 months before death, with a sharp decline in the values in 3 months leading up to their death. Therefore, patients and their families experiencing HSUV changes because of worsening disease conditions may experience difficulty coping with changing circumstances. In other words, our results supported the importance of advanced care planning and end-of-life communication in patients with MBC, as demonstrated by Sagara et al. [24].

We also examined the point estimates of HSUVs among the three groups (Figure 3). We observed an HSUV decline (disutility value) of approximately −0.1 for HSUVs in the PBC and MBC (survivors) groups and approximately −0.2 for those in the PBC and MBC (deceased) groups. A disutility value of approximately −0.1 was observed for the MBC (survivors) and MBC (deceased) groups. Therefore, HSUVs decreased with the progression of the clinical stage. Wang et al. [25] reported that HSUVs significantly decreased with increased breast cancer clinical stages (0.789, 0.793, 0.774, and 0.686 in stages I, II, III, and IV, respectively; *p* < 0.001), thereby producing disutility values of –0.103 for stage IV vs. stage I. However, the aforementioned results were consistent with those of our study. This concordance can be attributed to the racial concordance of the Asian cohort, considering the study subjects in Wang et al. were Chinese, and the methodological concordance of using the EQ-5D to validate HSUVs. For example, Hildebrandt et al. used the EQ-5D to examined a German cohort with race and reported no difference in median HSUVs between earlier (primary, non-MBC) and later (MBC) disease stages (both 0.887 based on EQ-5D-3L) [26]. In contrast, Chie et al. [27] performed a study of HSUVs using the direct method of TTO and SG in a Taiwanese breast cancer cohort of Asians. They assessed HSUVs via an expert panel (*n* = 31), thus revealing higher disutilities for later (metastatic) vs. earlier (localised) breast cancer stages using both SG (−0.750) and TTO (−0.642) approaches. In other words, the disutility values for different disease conditions can vary several-fold depending on the method of assessment. Thus, the disutility values of the dissimilar methods for assessing HSUVs differed significantly, despite including similar races. Therefore, HSUVs in patients with breast cancer should be considered only after an adequate confirmation of their ethnicity and the method of assessment (direct or indirect methods, such as EQ-5D).

Our study had some limitations. First, our study cohort was collected from a single centre in Japan, and the sample size was small, which may have resulted in biased inclusion. For example, because of the lack of racial diversity in our study, our findings may not be applicable to racial contexts of other geographical regions. Therefore, we carefully compared our findings with those of a systematic review of HSUVs in patients with breast cancer. Our findings were consistent with those of previous studies, despite the bias. Second, our research design focused on HSUVs in terminally ill patients with breast cancer 6 and 3 months before their death. We observed a sharp decline in HSUVs 3 months before death. In the future, studies should capture more comprehensive changes by examining patients at detailed time points to examine the HSUVs of those with breast cancer at the end of life.

## 5. Conclusions

This was the first study to describe the decline in HSUVs over time in patients with end-stage breast cancer shortly before their death. The MBC (deceased) group demonstrated the largest decrease in HSUVs, with an RMSE of 0.175. Patients with end-stage breast cancer had well-controlled HSUVs of 0.66, 3 months before their death, with a sharp decline in HSUVs in the 3 months leading up to death.

## Figures and Tables

**Table 1:** Clinical characteristics in patients with primary breast cancer.

Variable Patients	(*n* = 137)	
**Age (years), Mean ± SD (Range)**		**56.0 ± 11.16 (32–86)**
**Histopathological types**	***n***	**%**
Ductal carcinoma in situ	13	9.6
Invasive ductal carcinoma	102	75.0
Invasive lobular carcinoma	11	8.1
Others	10	7.3
**Breast cancer subtypes**	***n***	**%**
HR+/HER2– (luminal)	102	74.5
HR–/HER2+ (HER2)	7	5.1
HR+/HER2+ (luminal–HER2)	18	13.1
HR–/HER2− (triple-negative)	10	7.3
**Stage**	***n***	**%**
0	13	9.5
І	59	43.0
ІІA	36	26.3
ІІB	17	12.4
ІІІA	6	4.4
ІІІB	3	2.2
ІІІC	3	2.2

Abbreviations: SD, standard deviation; HR, hormone receptor; and HER2, human epidermal growth factor receptor 2.

**Table 2:** Clinical characteristics in patients with metastatic breast cancer.

Variable Patients	(*n* = 44)	
**Age (years), Mean ± SD (Range)**		**57.4 ± 11.6 (29–80)**
**Breast cancer subtypes**	***n***	**%**
HR+/HER2– (luminal)	30	68.1
HR–/HER2+ (HER2)	4	9.1
HR+/HER2+ (luminal–HER2)	4	9.1
HR–/HER2− (triple-negative)	5	11.1
Unknown	1	2.3
**Number of metastatic organs**	***n***	**%**
1	19	46.3
2	7	17.1
≥3	15	36.6
**Potentially life-threatening organ metastases (liver, lung, brain)**	***n***	**%**
+	27	61.4
−	17	38.6
**Metastatic organs (including duplicates)**	***n***	**%**
Liver	13	29.5
Lung	17	38.6
Brain	4	9.1
Bone	16	36.4
Distant LNs	17	38.6
Breast/skin	22	50.0

Abbreviations: SD, standard deviation; HR, hormone receptor; HER2, human epidermal growth factor receptor 2; and LN, lymph node.

## Data Availability

The data presented in this study are available upon request from the corresponding author. The data are not publicly available owing to patient privacy.
